# The Causes of the Failure of Gold as a Filling Material

**Published:** 1884-03

**Authors:** A. A. Blount

**Affiliations:** Geneva, Switzerland.


ARTICLE V.

### THE CAUSES OF THE FAILURE OF GOLD AS A FILLING MATERIAL.

BY DR. A. A. BLOUNT, GENENA, SWITZERLAND.

Read before the American Dental Society of Europe, at Cologne.

Ever since the "new departure" sprang into existence it has been a constant study to ascertain why gold had so suddenly become an unsafe material for preserving teeth. Had the profession at large exercised judgment and discrimination in the use of the various preparations of gold, the "new departure" would never had existed, for it is the out-growth of the indiscriminate use of heavy and extra cohesive foils, and the lack of system in the preparation of cavities. Thousands of teeth have been lost at the hands of men who stand high in the profession, and yet gold must take the blame, and not he who manipulates it. It is not to be wondered at that this outcry against gold should have gained the proportions it has, when we see teeth almost hopelessly decayed, that were once beautifully and elegantly filled. When they left the hands of the operator they were jewels in more senses than one, but alas, how soon the fell destroyer, decay, insinuated itself around the margins of that beautiful work of art, the possessor of which rested in perfect security in the belief that the operation was perfection, until warned by twinges of pain he sought the services of the most skillful dentist, who, upon examination found extensive decay going on silently but surely at the cervical and lateral borders. The dentist is astonished that such beautiful operations from the hands of such eminent men should so soon fail.

Is it to be wondered at that his faith in the preservative qualities of gold should be shaken? He is constrained to believe that some plastic material might have saved those teeth better than gold. Very true, but why should gold be condemned when the operator is at fault, who, placing too much reliance upon his manipulative skill does not stop to reflect, but goes on day by day committing the same error, until, seeing his own failures, he concludes that gold is not the best material after all for preserving teeth? It is thus that the "new departure" has gained proselytes. If those in the profession who have abandoned and condemned the use of gold as a filling material, and even brought chemistry and electricity to substantiate their opinions, had spent as much time in trying to discover the causes of their failures as they did in filling glass tubes with amalgams and kindred materials, they would to-day be saving more teeth with gold than they ever will with plastic fillings.

I shall mention a few of the causes which, in my judgment, produce the greatest number of failures.

1st. *The lack of a proper system in the formation of cavities.*

No preparation of gold can be perfectly adjusted to the walls and borders of a badly formed cavity. The introduction and condensing of gold is a simple and easy operation; any dentist of ordinary manipulative ability can make a good filling in a properly prepared cavity, while on the other hand, no dentist however skillful he may be can make a good filling in an imperfectly prepared one. The system of making retaining pits, so much in vogue, is a dangerous one, no matter where they may be located, as they are insecure and do not always answer the purpose for which they were intended. One who is in the habit of relying upon them for holding the foundation of his filling, is too apt to neglect more important considerations in the formation of the cavity. Aside from the danger of encroaching upon the pulp on the one hand, and drilling through the gum on the other, the gold is apt to move with every blow of the mallet, perhaps not perceptibly, but sufficiently to destroy the perfection of the filling.

Another frequent cause of failure is; *using heavy foil where it should not be used, l. c.*, within the body of the filling and against the walls and borders. No doubt many of us have seen teeth filled with No. 120, and even heavier rolled gold, driven into small cavities in incisors and bicuspids with a mallet weighing from eight to ten ounces, while the operator cries out to his assistant, with every stroke of the mallet, "Harder! harder!!"

It would be just as consistent to place the patient's head under a "drop," put a solid piece of gold over the cavity, let the drop come down, and thus fill the tooth at one blow.

*Extra cohesive gold in too large pieces*, either in cylinders or pellets—crowded into the cavity without any system—with the one idea of filling up fast.

*Imperfect adaptation of the gold to the walls of the cavity.* The heavy and cohesive foil becoming hard by manipulation, folds upon itself leaving pits through which the buccal fluids penetrate, often to the very bottom of the cavity, and in a short time a dark line becomes apparent, and disintegration and decay of the enamel and dentine follow.

*Injudicious use of the mallet* is also one of the causes of our failures; too much and too hard malleting with serrated instruments, especially over the centre of large fillings, causes the gold to draw away from the borders, no matter how carefully it might have been adjusted in the beginning. Decay as a matter of course supervenes.

*The lack of proper instruments to condense the gold against the borders.* However carefully serrated instrument may be used, it will more or less mar the borders of the cavity. The sharp serrations coming in contact with the edges of enamel must inevitably leave their mark, and into these little pits the hard or heavy gold cannot be forced. In the process of finishing, small particles of gold fill up these pits, hiding from the operator the imperfections, and he is surprised to see the filling in a short time present such early signs of failure.

If a careful preparation of cavities and a judicious selection of various preparations of gold, with intelligent manipulation of that which in our judgment is best suited for each individual cavity can in any degree serve to lessen our failures, we should leave no method untried to make our operations more perfect, and thus wipe out the "new departure" from our midst, or confide it to the tender mercies of charlatans. In my judgment, in order to correct some of the failures mentioned above, we should adopt some systematic method of preparing cavities and of introducing and condensing the gold, follow up that system persistently until we become so expert in it that filling teeth shall be a work of pleasure rather than of labor.

In order to obtain the best results, that system must be based upon scientific mechanical principles. I consider the formation of the cavity by far the most important part of the operation in filling, and shall at some future meeting, if the Society desires, classify and describe my method of preparing cavities, at the same time presenting drawings of each class.—*Independent Practitioner.*

